# Gunshot Wound in the Head—Bullet Imbedded in Brain Substance—Death on Eleventh Day—Post Mortem

**Published:** 1880-02

**Authors:** D. A. K. Steele

**Affiliations:** Chicago


### Clinical Reports.

### NOTES FROM PRIVATE PRACTICE.

### ARTICLE VII.

*Gunshot Wound in the Head.—Bullet Imbedded in Brain-Substance.—Death on Eleventh Day.— Post Mortem.*

Henry K., aged 32, a robust, well-formed coachman, attempted suicide October 1st, 1879, by shooting himself through the head. He fired at the centre of his forehead, while lying on his back in bed, the aim being rendered more accurate by means of a small mirror which he held in his left hand, while the pistol, which carried a No. 32 ball, was held in his right hand. I saw him a few hours after the injury was received, and found him lying on his back in bed, perfectly conscious, with a bullet wound in the middle of the forehead, one inch above the level of the supra-orbital ridges and one-eighth of an inch to the right of the mesial line.

From the wound there had been copious hemorrhage, as his face, neck and pillows were covered with clotted blood. There was a circle, about one inch around the wound, powder-marked. A probe passed into the wound, downwards and to the right, about three inches, traversing the anterior lobe of the right hemisphere without encountering the bullet. A number of loose pieces of bone were felt by the probe as it was carried through the aperture in the skull.

The pupils were normal in appearance, and responded to light; no deviation in size; tongue readily protruded in any direction; special senses of audition, vision, gustation, olfaction and touch unimpaired; patient perfectly conscious and free from paralysis; pulse 84, moderately full; respirations 18; stated that just after he pulled the trigger he felt a sensation of numbness all over—

this lasted a few minutes, when he felt a deep-seated pain in both ears; blood escaped freely from the nose and mouth, and he vomited considerable blood. Water dressings were applied, and absolute rest and quiet enjoined. Gave potassium bromide, .50 grams every three hours.

8 P. M. Dr. A. Reeves Jackson saw him with me, and probed carefully for the ball, but was unable to detect it. It was decided, in the absence of any serious symptoms, to make no further search, but await developments.

October 2d, 9 A. M. Pulse 76, respiration 20. Rested well during the night, sleeping fifteen or twenty minutes at a time; pain in the ears much less; vomited once or twice; no bleeding from nose or throat; slight bloody discharge from wound.

12 M. Pulse 76. Diet to consist of milk, beef tea, oat-meal porridge, and gruel.

6. P. M. Pulse 72. Passed urine freely, natural in amount.

10 P. M. Pulse 70. Sleeping quietly, not a bad symptom.

October 3d, 9 A.M. Pulse 62; respiration 20; tongue clean; no fever; face and neck hyperæsthetic; slight shooting pains through the head, and soreness at the posterior margin of vertex; sero-sanguineous discharge from wound; picked out a small detached piece of bone.

2:30 P. M. Pulse 60. Slight œdema and ecchymosis of left eye-lid; circular slough three-fourths of an inch in diameter forming around wound. Ordered (ʒv) 20 gr. of Rochelle salts to move bowels.

7 P. M. Pulse 60, respiration 22. Dr. Jackson again visited the patient with me, and we deemed it best not to probe the wound, as there were no symptoms to indicate cerebral disturbance.

October 4th, 9 A. M. Pulse 60, respiration normal. Feels bright and cheerful; has a better appetite.

7 P. M. Pulse 64. Slight pain in head, back of left eye and towards the ear, for the past hour; attendant had allowed him to walk across the room, and get up to evacuate the bowels, contrary to directions.

9 P. M. Pain in the head has been steadily increasing in severity, is jumping or shooting in character, and referred to the temples and back of eyes; patient very restless and uneasy, crying out with pain; intense thirst; skin hot. Pulse 68. Increased dose of bromide to (℈i) 1.20 grams, and added (gtt. i) .05 c. c. tr. aconiti rad. every three hours.

11:30 P. M. Pulse 80. Gave medicine every two hours.

October 5th, 2:30 A. M. Pulse 60, full and regular; feels better; dressed wound and removed fragment of bone; some pulpified brain-matter escaped; no odor to discharges, as dressings have been carefully carbolized.

11 A. M. Was seen by Dr. Jackson; symptoms more favorable; procured rubber ice bag for his head to rest on; advised chloral hydrate to allay pain and procure sleep, (15 grs.) one gram as needed.

4 P. M. Pulse 62.

10 P. M. Pulse 60. Slept forty-five minutes after taking the chloral hydrate.

October 6th, 9 A. M. Pulse 64. Slept about five hours during the night; wound looks well.

2:30 P. M. Pulse 64. Both eyelids ecchymotic.

9 P. M. Pulse 84. Is drowsy and stupid; does not answer promptly when spoken to; pupils slightly contracted—respond to light; tongue heavily furred; eyelids agglutinated by mucus; has a feeling of soreness all over the head; no acute pain. As four grams (ʒi) of chloral had been taken during the afternoon, the drug was now discontinued.

October 7th, 9 A. M. Pulse 86. Had a comfortable night; removed two small detached pieces of bone from the wound.

2 P. M. Pulse 88, full and strong.

7 P. M. Pulse 86. Gave (gtt. i) .05 c. c. tr. aconiti rad. every hour.

October 8th, 8 A. M. Pulse 76. Was rather restless during the night; is perfectly conscious and hungry, taking milk, beef tea and oat-meal gruel freely.

2 P. M. Pulse 74. Resting quietly.

11:30 P. M. Pulse 72. Resting quietly; talks intelligently when addressed; expresses himself as feeling better; urinates regularly and easily, calling for a vessel when needed.

October 9th, 8:30 A. M. Pulse 74. Is bright and cheerful; feels well.

12 M. Had slight difficulty in swallowing; cervical muscles rigid; cannot turn his head from side to side.

9 P. M. Pulse 74. Muscles of deglutition paralyzed; lies on left side, face drawn to the left.

October 10th, 7 A. M. Pulse 130, respiration 54. Left hemiplegia; case hopeless.

9 A. M. Passed urine involuntarily.

3 P. M. Pulse 140. Thready, muscular twitching.

6 P. M. Pulse 136, respiration 50. Had an epileptiform convulsion.

8 P. M. Has had three more convulsions; right eye turned in; left not affected; left bronchial tubes filled with mucus; used catheter, removing 700 c. c. (22 oz.) of ammoniacal urine.

October 11th, 7 A. M. Patient died, having had several convulsions during the night. Autopsy two hours later, in the presence of Drs. A. R. Jackson, J. C. Moore and nurses Russ and Brady.

*Post-mortem Appearance.*—The autopsy was made on Saturday morning, October 11, 1879, at 8:50 o'clock.

Body slightly emaciated; no rigor mortis.

An external pistol-ball wound in forehead about an inch and a half above the root of the nose, and slightly to the right of the median line.

The scalp was then incised from ear to ear across the vertex, and the flap drawn forward over the face. Upon the inner side of the scalp an ecchymosis of four inches in diameter existed around the wound.

The opening in the skull caused by the ball was of slightly crescentic shape, about three-quarters of an inch wide, by half an inch from above downward. No fracture lines were apparent upon the outer table extending from the opening. A small chip of lead was found attached to the roughened bone at the site of the wound. Upon removing the skull-cap, the right frontal sinus was found involved. Its walls were somewhat shattered by the passage of the ball, and the sinus itself contained a little bloody matter upon its membrane, and some small pieces of shattered bone. The blood which had issued from the patient's nose and mouth in the early history of the case had doubtless found its way through this sinus to the nasal cavity and pharynx.

The vitreous table, from its anatomical construction and its situation, suffered most. The brittleness of the bone-tissue, and the nearly spent force of the ball, contributed in causing the internal opening to be larger than the external. Most of the pieces of bone which had been removed through the wound belonged to this table, and now were found two plates of the vitreous layer, each about a half-inch in diameter, adherent by lymph to the dura mater.

The dura mater presented no specially abnormal appearance, save the adherent scales of bone alluded to, and the opening caused by the ball, around which there was a limited inflammatory condition, and softened and disorganized brain substance was issuing from the orifice. Upon removing the dura mater of the right cerebrum, a small quantity of bloody serum, mixed with pus, was found beneath, and patches of pus were found over the pia mater, chiefly along the course of the vessels—the vessels themselves being slightly congested.

The left cerebrum was not affected.

In making sections of the brain substance, the same congested condition of the vessels was apparent. The right ventricle contained a considerable quantity of healthy fluid.

The ball, circular and flattened, was found lodged within the anterior lobe of the right cerebrum, about three-quarters of an inch deep on a horizontal line, and slightly to the right of the longitudinal fissure.

Evidently the cause of death was traumatic inflammation of the brain.

This case is reported not that it presents any strikingly new features of diagnosis or treatment, but as a contribution to the pathology of brain lesions. This case clearly shows that a comparatively large foreign body may be imbedded in the brain substance for ten days without giving rise to any symptoms of particular gravity, such as impairment of the special senses or paralysis. It also favors Ferrier's view of cerebral localization, as here we had extensive destruction of the middle convolutions of the right frontal lobe, not followed by paralysis of motion or sensation, the functions of these convolutions being concerned in the manifestation of intellectual acts. Later, as a result of inflammation and consequent extension of the lesion to the basis of these convolutions, to the area presiding over motion, we had muscular rigidity of the opposite half of body, followed by motor paralysis; also oculo-motor paralysis, with turning of the head to the opposite side.

D. A. K. Steele, m.d.

Chicago.

